# Supplementary material for: Model selection may not be a mandatory step for phylogeny reconstruction
Source: Nat Commun. 2019 Feb 25;10:934. doi: 10.1038/s41467-019-08822-w (PMC6389923; doi:10.1038/s41467-019-08822-w)
Supplement: Supplementary file 1 — Supplementary Information [file 41467_2019_8822_MOESM1_ESM.pdf]

Supplementary Information

Model selection may not be a mandatory step for phylogeny reconstruction

Abadi et al.

This PDF file includes:

Supplementary Figures 1-5
Supplementary Table 1

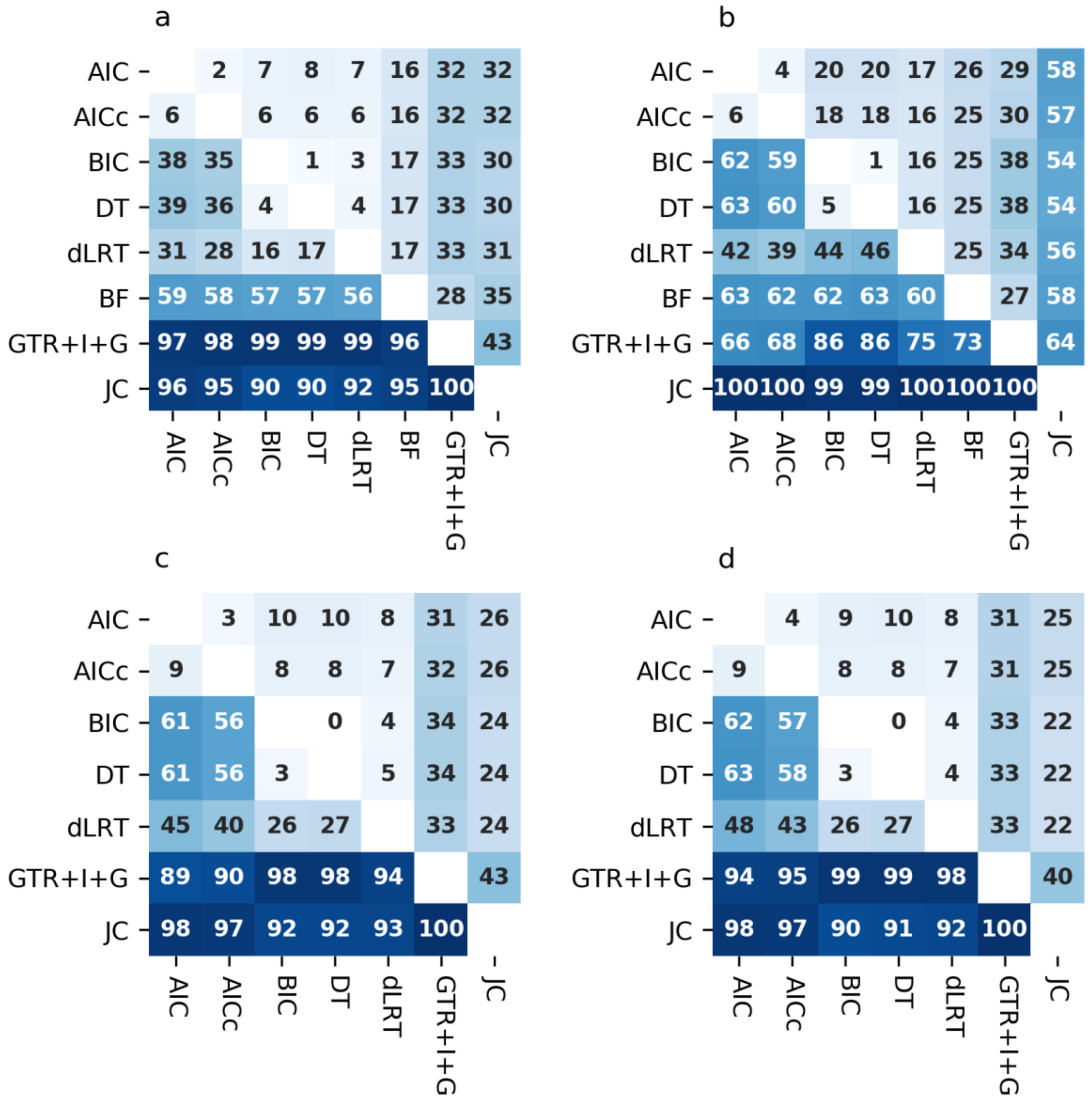

Supplementary Figure 1. Pairwise distances between the trees inferred by the evaluated strategies for a subset on which BF was computed. The number within each cell represents the percentage of discrepancies between the two strategies at the row and column. The upper right triangles represent the percentage of different topologies and the lower left triangles represent different branch length estimation. The matrices represent the following datasets: (a) simulation set c_0, (b) the empirical set, (c) simulation set c_1, and (d) simulation set c_2. The percentages were computed over a subset of 1,500 datasets for which BF was computed. For the analysis over the complete set, see Fig. 1.

a. Simulation set c_0

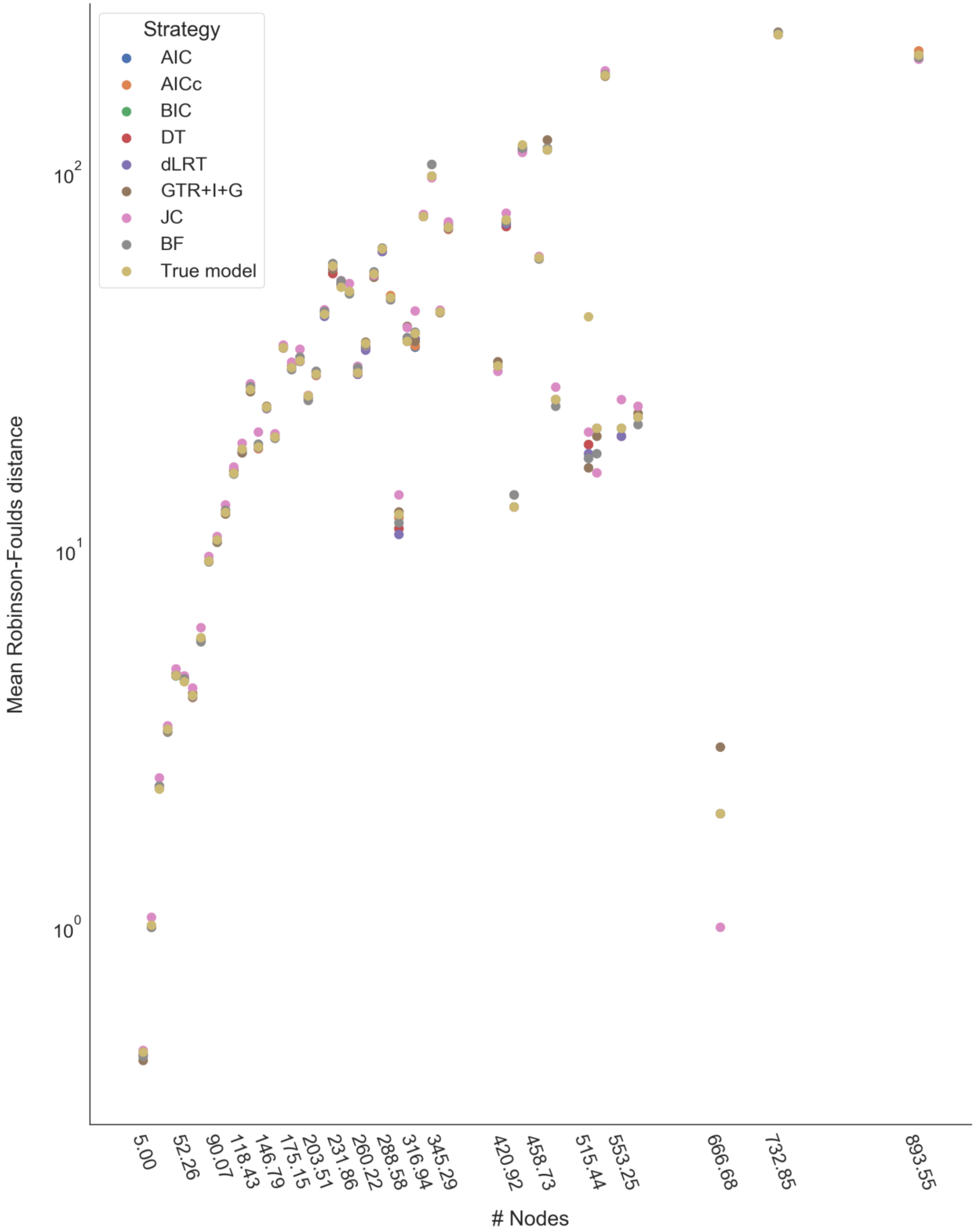

b. Simulation set c_1

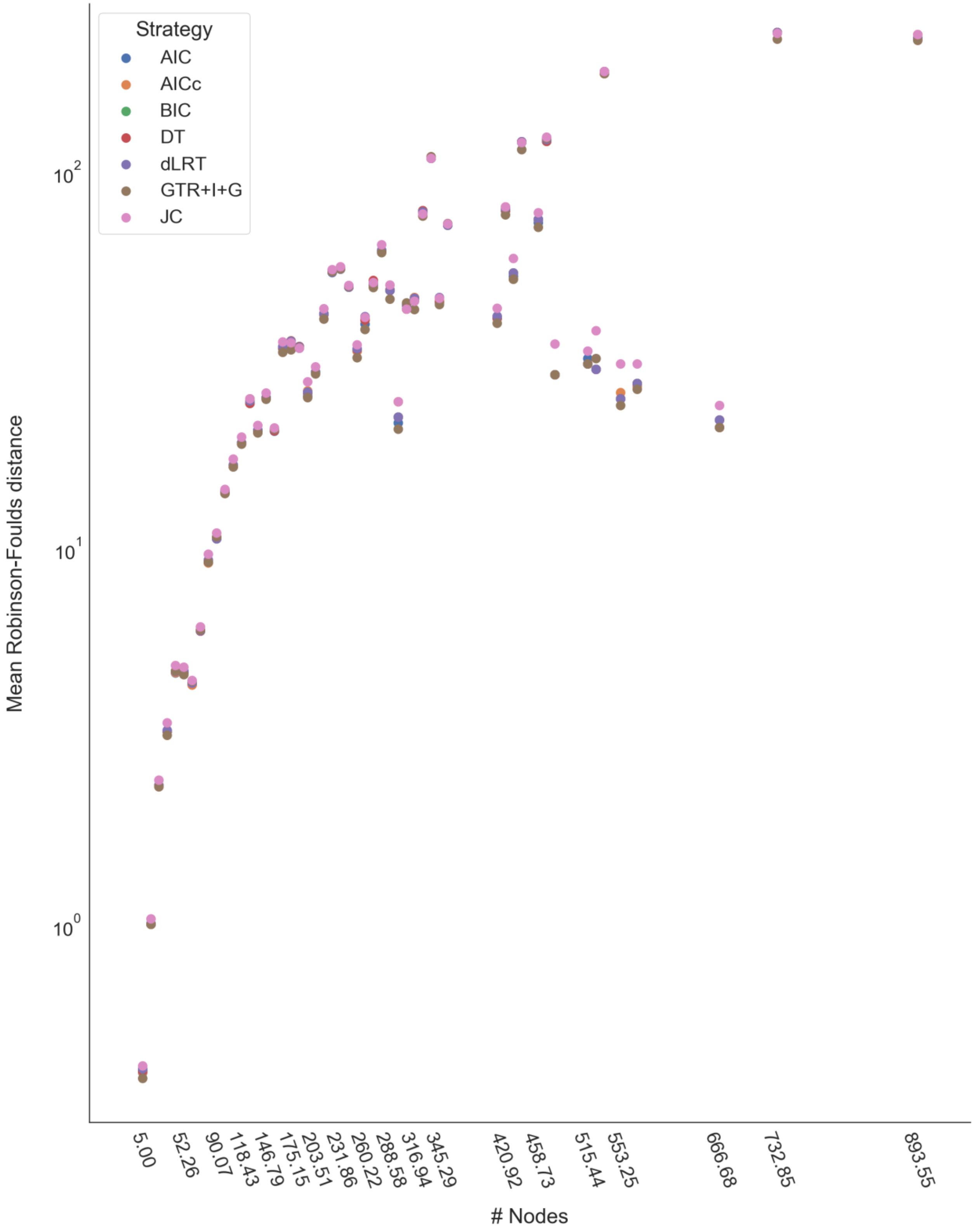

c. Simulation set c_2

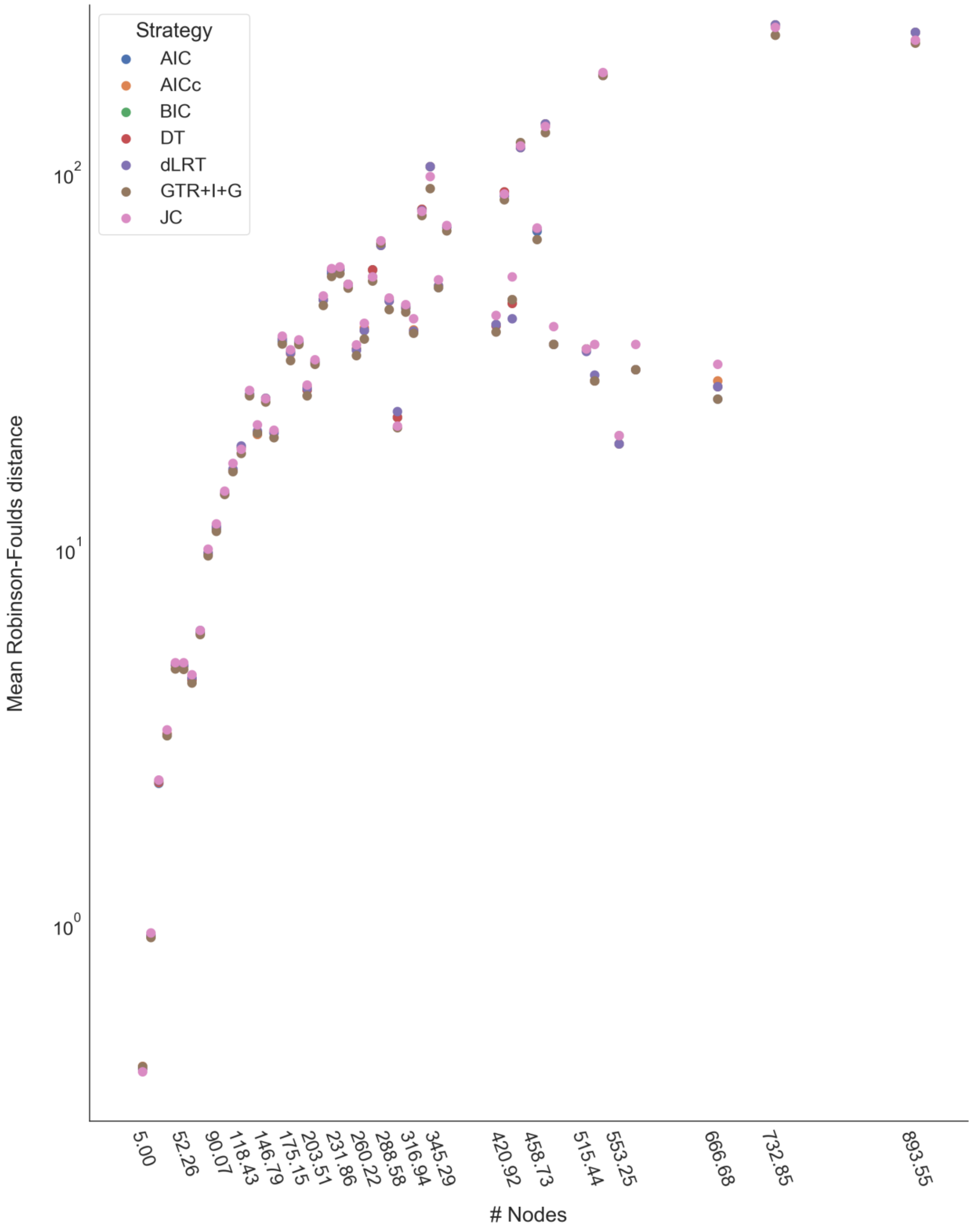

d. Simulation set c_3

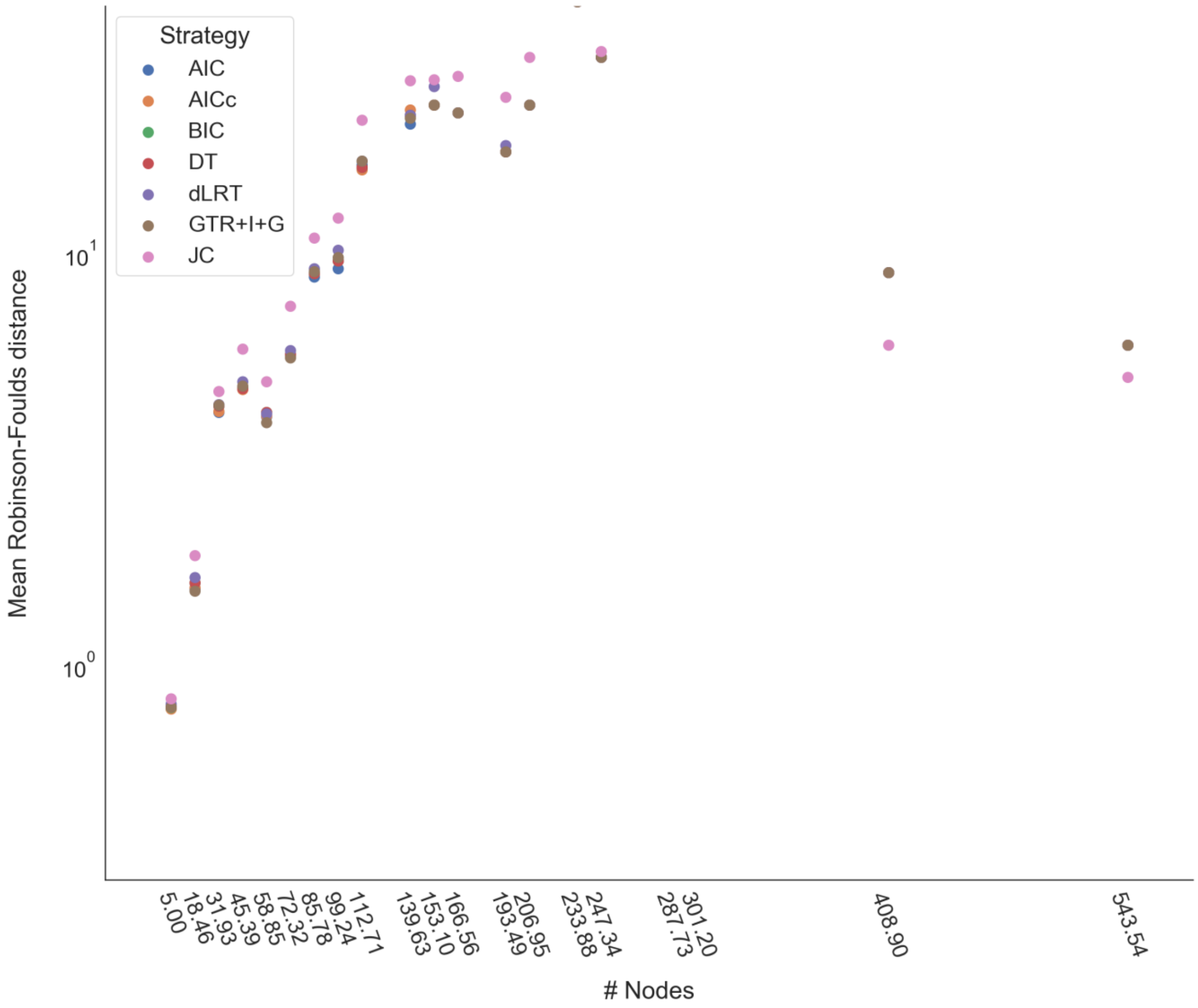

Supplementary Figure 2. Robinson-Foulds distances of the selected trees from the true trees for increasing tree size. The datasets of each simulation set were binned according to the number of nodes in the trees (x axis). For each dataset and strategy (either criterion, the GTR+I+G model, the JC model, or the true model used for its simulation; see legend), the Robinson-Foulds distance between the reconstructed and true tree was computed. The y axis represents the mean over the distances of the datasets in each bin in log scale (for numeric data, see Supplementary Data 2). For the mean of ranks of the distances across all datasets, see Table 3. Equal-width bins were determined according to Scott's normal reference rule which minimizes the integrated mean squared error of the density estimate.

a. Simulation set c_0

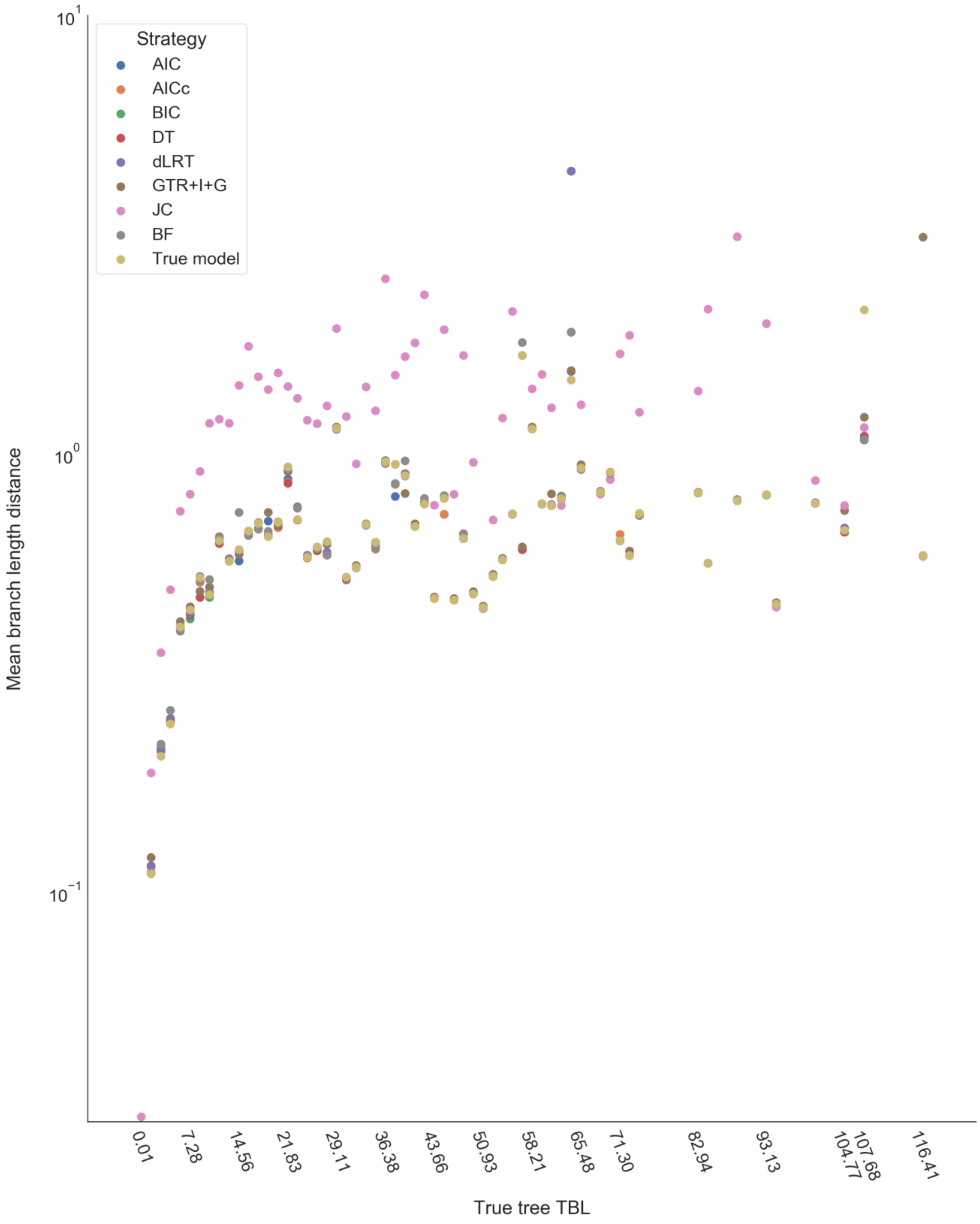

b. Simulation set c_1

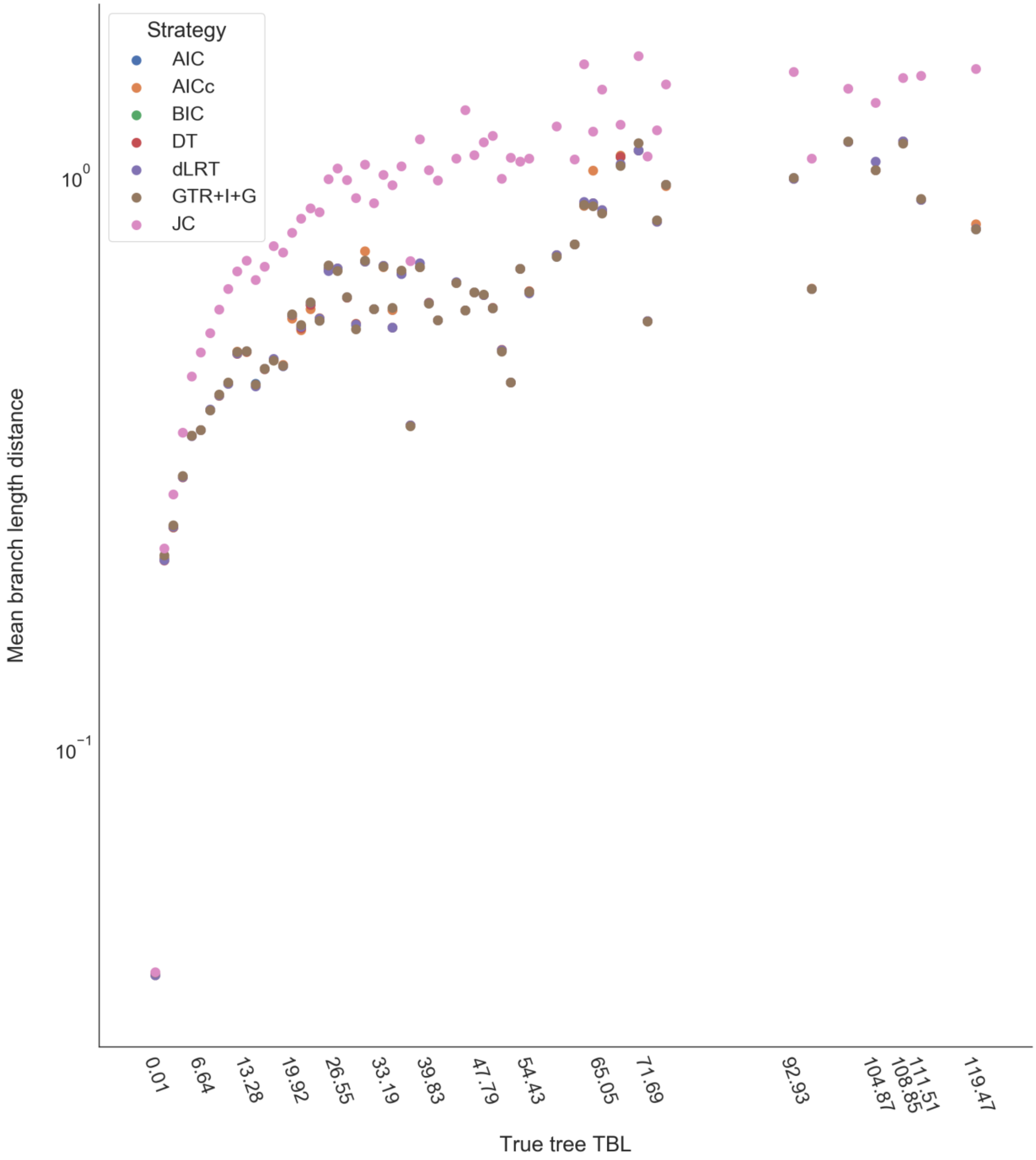

c. Simulation set c_2

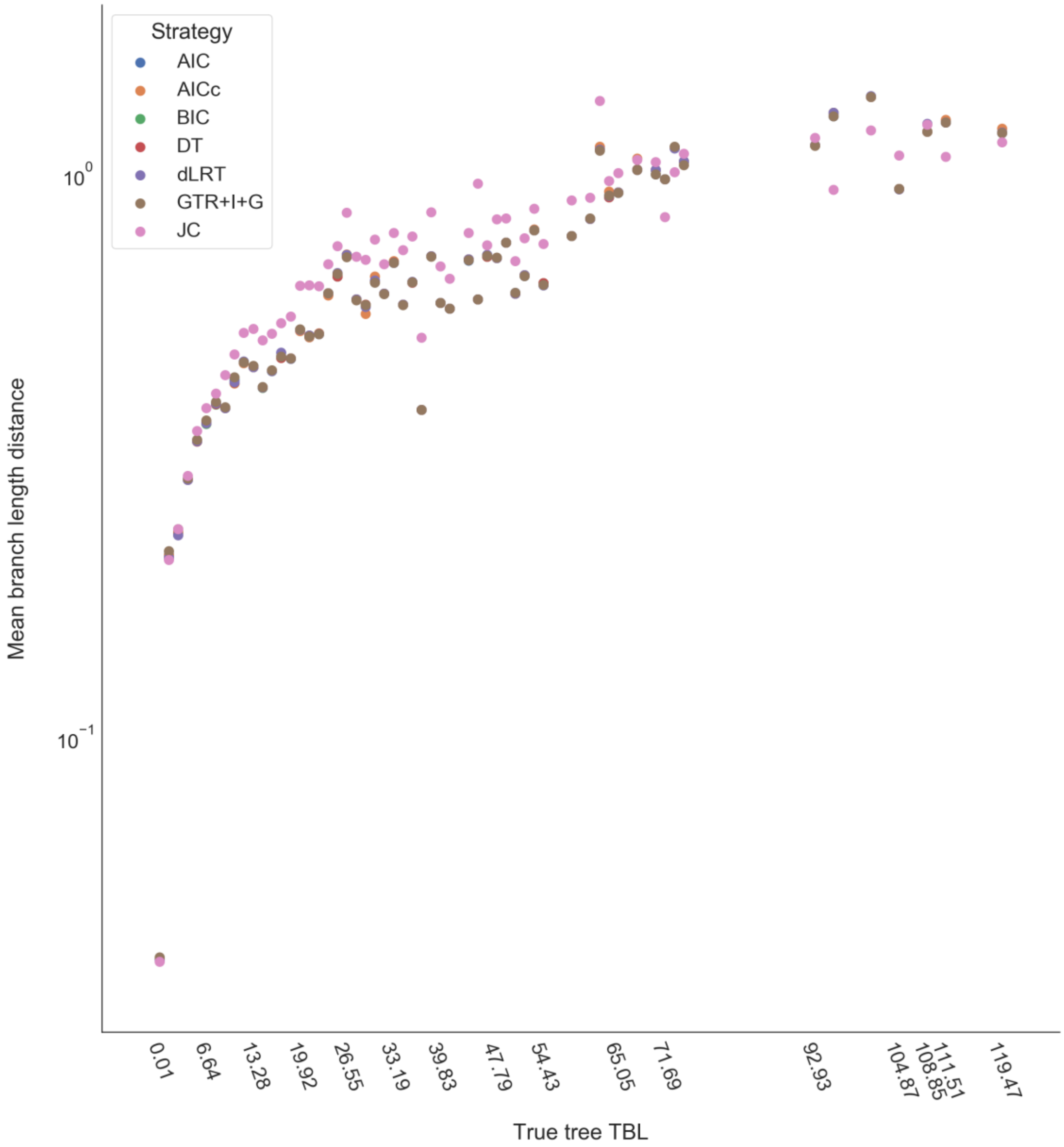

Supplementary Figure 3. Branch length distances of the selected trees from the true trees for increasing tree size. The datasets of each simulation set were binned according to the Total Branch Lengths of the true trees (x axis). For each dataset and strategy (either criterion, the GTR+I+G model, the JC model, or the true model used for its simulation; see legend), the branch length distance between the reconstructed and true tree was computed. The y axis represents the mean over the branch length distances of the datasets in each bin in log scale (for numeric data, see Supplementary Data 3). For the mean of ranks of the distances across all datasets, see Table 4. Equal-width bins were determined according to Scott's normal reference rule which minimizes the integrated mean squared error of the density estimate.

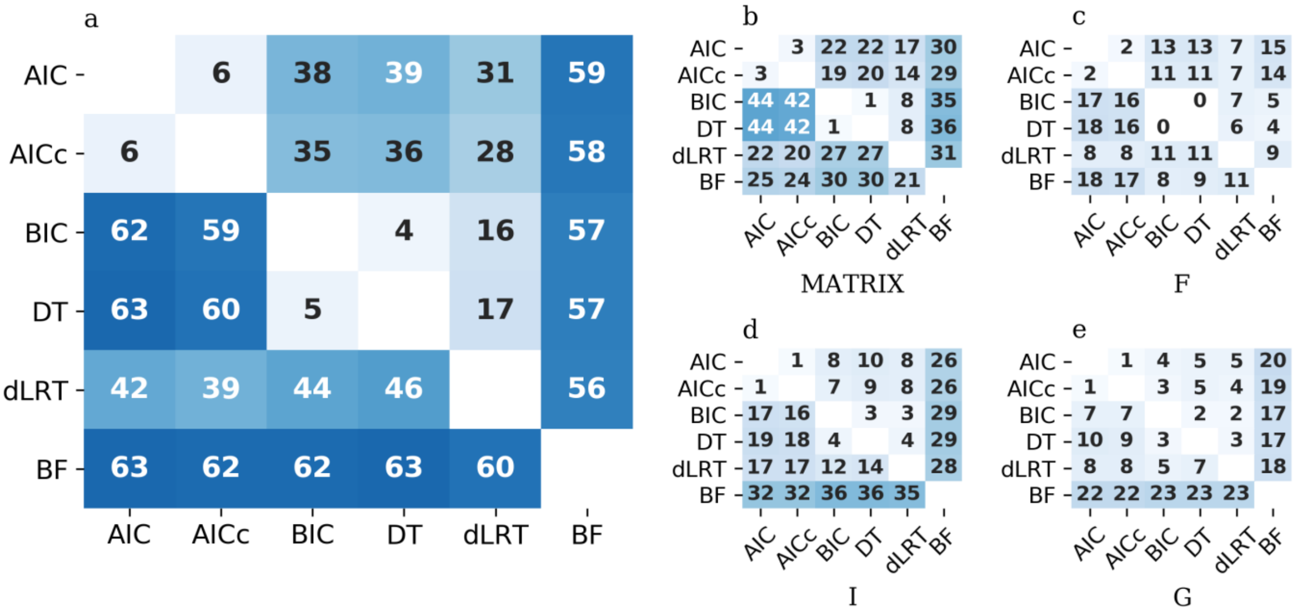

Supplementary Figure 4. Incongruency over the selections of models over the simulated and empirical datasets filtered to the subset on which BF was computed. The matrices represent the percentage of the data that a pair of criteria in the corresponding column and row disagreed on. (a) represents the disagreement over the entire model (one of 24 models) while (b-e) represent disagreement over components of the models: (b) matrix of one, two, or six rate parameters, (c) the inclusion of the F parameters (unequal base frequencies), (d) the inclusion of the I parameter (proportion of invariable sites), (e) the inclusion of the G parameter (heterogeneous rates across sites following the gamma distribution). The percentages below and above the left diagonal represent the percentage of dissimilarities over empirical data and data simulated with the common models, respectively. The percentages were computed over a subset of 1,500 datasets for which BF was computed. For the analysis over the complete set, see Fig. 3.

a

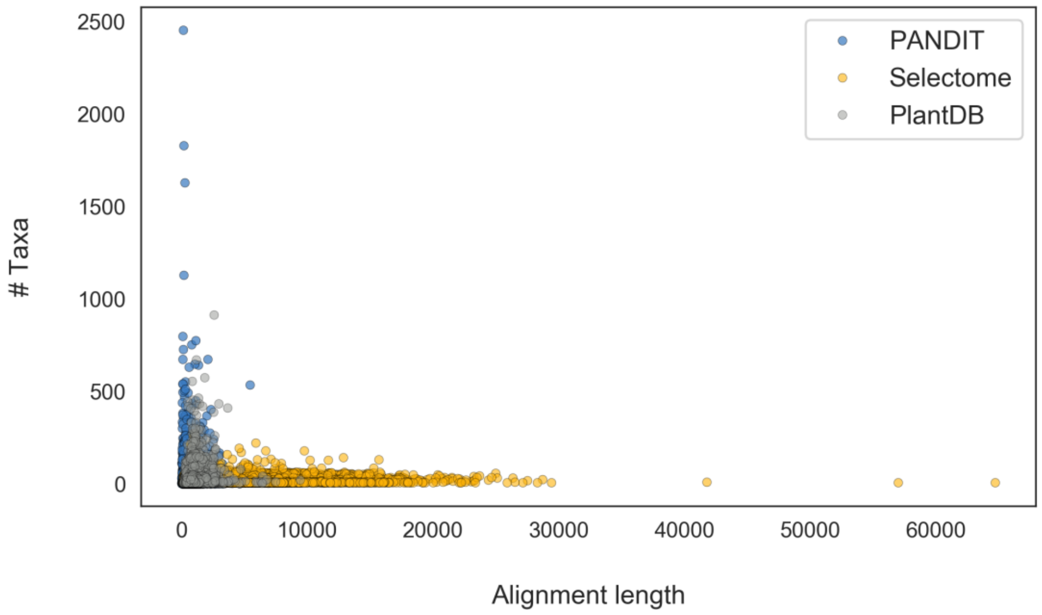

b

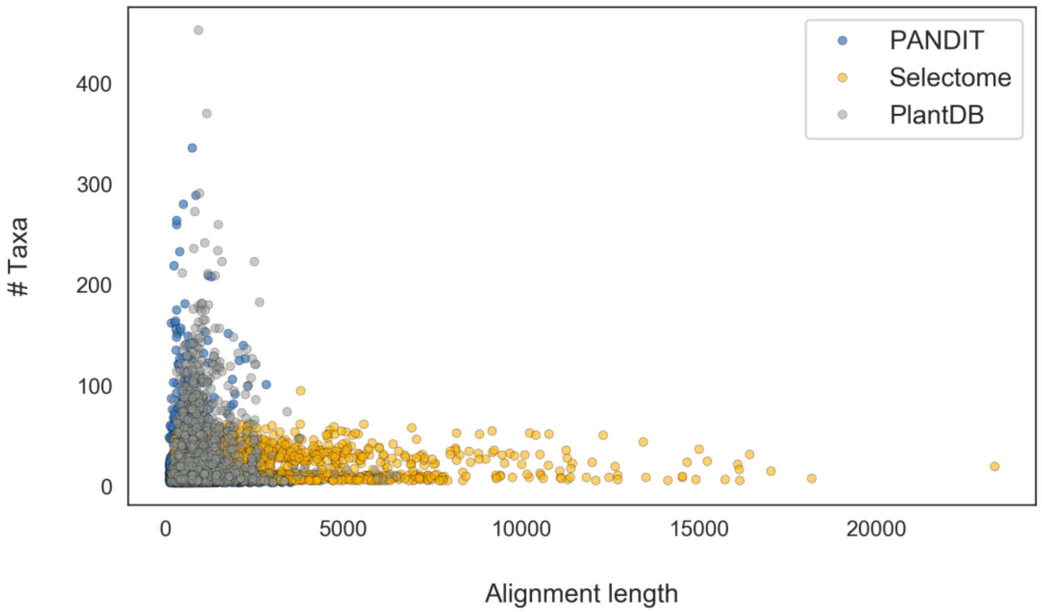

Supplementary Figure 5. Data distribution. The distribution of the samples in the three databases: PlantDB[1], Selectome[2], and PANDIT[3] in terms of alignment length (x axis) and number of sequences (y axis). (a) Distribution of all the datasets that are included in these databases. (b) Distribution of the 7,200 samples that were sampled for this study.

Supplementary Table 1. Ancestral sequence reconstruction average distances between the reconstructed root sequence and the true one across different scales for the various reconstruction strategies. Average fraction of nucleotides that were different between the true and the reconstructed sequence according to the best models by each of the criteria and consistently using JC or GTR+G. The different tree scales represent the extent of sequence divergence (see Methods). (a) The analysis over the simulated dataset c_0; (b) The analysis over datasets simulated with complex model c_2. For visual representation, see Fig. 2.

(a)

Strategy / Tree scale	AIC	AICc	BIC	DT	dLRT	GTR+G	JC
**original**	0.005041	0.005042	0.005058	0.005058	0.005049	0.005094	0.00537
**0.08**	0.000549	0.000552	0.000539	0.000539	0.000554	0.000562	0.000583
**0.16**	0.00164	0.001638	0.001636	0.001636	0.001644	0.001653	0.001742
**0.27**	0.003675	0.003671	0.003679	0.003679	0.003669	0.003667	0.003947
**0.53**	0.010736	0.010738	0.010674	0.010674	0.010737	0.010812	0.011727
**1.19**	0.033045	0.033013	0.033098	0.033098	0.03298	0.033204	0.036123
**2.18**	0.067852	0.06787	0.068118	0.068118	0.068081	0.068754	0.074616
**3.5**	0.112168	0.112232	0.113056	0.113056	0.113446	0.113661	0.121379
**5.18**	0.161564	0.161853	0.163255	0.163259	0.164355	0.162623	0.173381
**9.5**	0.254285	0.254361	0.253868	0.253828	0.254799	0.254926	0.265364

(b)

Strategy / Tree scale	AIC	AICc	BIC	DT	dLRT	GTR+G	JC
**original**	0.003678	0.003679	0.003677	0.003677	0.003668	0.003665	0.003736
**0.08**	0.000314	0.000314	0.000312	0.000312	0.00031	0.000313	0.000309
**0.16**	0.000885	0.000886	0.000872	0.000872	0.00087	0.000881	0.000858
**0.27**	0.0021	0.0021	0.002082	0.002082	0.00208	0.002092	0.002113
**0.53**	0.006938	0.006934	0.006924	0.006924	0.00694	0.0069	0.007003
**1.19**	0.025978	0.02597	0.026041	0.026039	0.026061	0.02599	0.026421
**2.18**	0.063905	0.063907	0.06373	0.063729	0.063701	0.064507	0.06435
**3.5**	0.124287	0.124325	0.124302	0.124304	0.124516	0.124777	0.124912
**5.18**	0.201487	0.201441	0.201777	0.201711	0.202367	0.200748	0.206562
**9.5**	0.340464	0.340608	0.339835	0.339834	0.339781	0.341459	0.343649

References:

1. Glick, L., Sabath, N., Ashman, T.-L., Goldberg, E. & Mayrose, I. Polyploidy and sexual system in angiosperms: Is there an association? *Am. J. Bot.* **103,** 1223–35 (2016).
2. Moretti, S. *et al.* Selectome update: Quality control and computational improvements to a database of positive selection. *Nucleic Acids Res.* **42,** D917–D921 (2014).
3. Whelan, S., De Bakker, P. I. W., Quevillon, E., Rodriguez, N. & Goldman, N. PANDIT: an evolution-centric database of protein and associated nucleotide domains with inferred trees. doi:10.1093/nar/gkj087
