# Supplementary material for: Model selection may not be a mandatory step for phylogeny reconstruction
Source: Nat Commun. 2019 Feb 25;10:934. doi: 10.1038/s41467-019-08822-w (PMC6389923; doi:10.1038/s41467-019-08822-w)
Supplement: Supplementary file 3 — Description of Additional Supplementary Files [file 41467_2019_8822_MOESM3_ESM.pdf]

Description of Additional Supplementary Files

File Name: Supplementary Data 1
Description: Current model selection practices. The table presents the criterion of choice for 300 phylogenetic studies that used jModelTest (Darriba et al., 2012) for model selection during 2017-2018, downloaded from Web of Science (https://clarivate.com/products/web-of-science/).

File Name: Supplementary Data 2
Description: Averaged RF distance for all strategies across bins of increasing tree sizes. The datasets of each simulation set were binned according to the number of nodes in the trees. For each dataset and strategy (columns; the criteria, the GTR+I+G model, the JC model, or the true model used for its simulation), the Robinson-Foulds distance between the reconstructed and true tree was computed and averaged over all datasets within each bin. Equal-width bins were determined according to Scott's normal reference rule which minimizes the integrated mean squared error of the density estimate. Each sheet corresponds to the analysis of each simulation set c0-c3. For a visual representation, see Supplementary Figure 2.

File Name: Supplementary Data 3
Description: Averaged branch lengths distance for all strategies across bins of increasing tree sizes. The datasets of each simulation set were binned according to the total branch lengths (TBL) of the true trees. For each dataset and strategy (columns; the criteria, the GTR+I+G model, the JC model, or the true model used for its simulation), the branch lengths distance between the reconstructed and true tree was computed and averaged over all datasets within each bin. Equalwidth bins were determined according to Scott's normal reference rule which minimizes the integrated mean squared error of the density estimate. Each sheet corresponds to the analysis of each simulation set c0-c2. For a visual representation, see Supplementary Figure 3.
