# Supplementary material for: Model selection may not be a mandatory step for phylogeny reconstruction
Source: Nat Commun. 2019 Feb 25;10:934. doi: 10.1038/s41467-019-08822-w (PMC6389923; doi:10.1038/s41467-019-08822-w)
Supplement: Supplementary file 7 — Reporting Summary [file 41467_2019_8822_MOESM7_ESM.pdf]

nature research

Corresponding author(s): Mayrose Itay, Pupko Tal

Last updated by author(s): Jan 22, 2019

Reporting Summary

Nature Research wishes to improve the reproducibility of the work that we publish. This form provides structure for consistency and transparency in reporting. For further information on Nature Research policies, see Authors & Referees and the Editorial Policy Checklist.

Statistics

For all statistical analyses, confirm that the following items are present in the figure legend, table legend, main text, or Methods section.

n/a	Confirmed	
☐	☒	The exact sample size (*n*) for each experimental group/condition, given as a discrete number and unit of measurement
☒	☐	A statement on whether measurements were taken from distinct samples or whether the same sample was measured repeatedly
☐	☒	The statistical test(s) used AND whether they are one- or two-sided<br>*Only common tests should be described solely by name; describe more complex techniques in the Methods section.*
☒	☐	A description of all covariates tested
☐	☒	A description of any assumptions or corrections, such as tests of normality and adjustment for multiple comparisons
☐	☒	A full description of the statistical parameters including central tendency (e.g. means) or other basic estimates (e.g. regression coefficient) AND variation (e.g. standard deviation) or associated estimates of uncertainty (e.g. confidence intervals)
☐	☒	For null hypothesis testing, the test statistic (e.g. F, t, r) with confidence intervals, effect sizes, degrees of freedom and P value noted<br>*Give P values as exact values whenever suitable.*
☐	☒	For Bayesian analysis, information on the choice of priors and Markov chain Monte Carlo settings
☒	☐	For hierarchical and complex designs, identification of the appropriate level for tests and full reporting of outcomes
☒	☐	Estimates of effect sizes (e.g. Cohen's d, Pearson's r), indicating how they were calculated

Our web collection on statistics for biologists contains articles on many of the points above.

Software and code

Policy information about availability of computer code

Data collection	Datasets of multiple sequence alignments were sampled from three databases: (1) PlantDB (Glick et al., 2016), (2) Selectome version v06 (Moretti et al., 2014), (3) PANDIT version 17.0 (Whelan et al., 2003).
Data analysis	The code for this study was written in python version 3.6. Computation of likelihood and parameter estimates, model selection, simulations, and tree comparison were executed using the applications under the following versions: PhyML 3.0, RevBayes 1.0.6, PAML 4.8, jModelTest 2.1.7, Rate4site 3.2, INDELible 1.03, Treedist 1.0, and TreeCmp 1.0-b291.

For manuscripts utilizing custom algorithms or software that are central to the research but not yet described in published literature, software must be made available to editors/reviewers. We strongly encourage code deposition in a community repository (e.g. GitHub). See the Nature Research guidelines for submitting code & software for further information.

Data

Policy information about availability of data

All manuscripts must include a data availability statement. This statement should provide the following information, where applicable:

- Accession codes, unique identifiers, or web links for publicly available datasets
- A list of figures that have associated raw data
- A description of any restrictions on data availability

The datasets contained within the empirical set and the four simulated sets (c0-c3) have been deposited in Open Source Framework (OSF) with the identifier DOI 10.17605/OSF.IO/T3PF2.

Field-specific reporting

Please select the one below that is the best fit for your research. If you are not sure, read the appropriate sections before making your selection.

☐ Life sciences ☐ Behavioural & social sciences ☒ Ecological, evolutionary & environmental sciences

For a reference copy of the document with all sections, see nature.com/documents/nr-reporting-summary-flat.pdf

Ecological, evolutionary & environmental sciences study design

All studies must disclose on these points even when the disclosure is negative.

Study description	Model selection is a time-intensive step of molecular phylogenetic analysis. Here, Abadi, Azouri and colleagues show that all model selection criteria lead to similar inferences, and that for topology and ancestral sequence reconstruction, using the GTR+I+G model is as accurate.
Research sample	For a preliminary assessment of the discrepancies between the different model selection criteria, we assembled a database encompassing 7,200 multiple sequence alignments (MSAs), 2,400 from each of the following three databases: PlantDB, Selectome, and PANDIT. The datasets in PlantDB were generated as described in Glick et al., such that each MSA contains sequences belonging to a single plant genus and a potential outgroup. These MSAs contain between 2 to 912 species and span over 115 to 9,417 aligned sites. The Selectome database includes codon alignments of species within four groups (Euteleostomi, Primates, Glires, and Drosophila), which extend from 6 to 257 sequences and 72 to 64,734 aligned sites. The PANDIT database includes alignments of protein sequences that extend from 2 to 2,453 sequences and 15 to 6,895 aligned sites. A subset of 2,400 datasets was randomly selected from each database, excluding alignments that contained fewer than four sequences, fewer than 100 alignment sites, or produced low total divergence (i.e., when the multiplication of the total branch length by the alignment length is lower than 10).
Sampling strategy	Sampling size was determined to produce statistical power and encompass the range of datasets sizes. Datasets were randomly sampled from each database.
Data collection	Data were downloaded from the online databases sources by S. Abadi, sampled and processed using python scripts.
Timing and spatial scale	Not relevant.
Data exclusions	Not relevant.
Reproducibility	Not relevant.
Randomization	Datasets were randomly selected from each database: 2400 from each one.
Blinding	Not relevant - data were obtained from online resources.

Did the study involve field work? ☐ Yes ☒ No

Reporting for specific materials, systems and methods

We require information from authors about some types of materials, experimental systems and methods used in many studies. Here, indicate whether each material, system or method listed is relevant to your study. If you are not sure if a list item applies to your research, read the appropriate section before selecting a response.

Materials & experimental systems

n/a	Involved in the study
☒	☐ Antibodies
☒	☐ Eukaryotic cell lines
☒	☐ Palaeontology
☒	☐ Animals and other organisms
☒	☐ Human research participants
☒	☐ Clinical data

Methods

n/a	Involved in the study
☒	☐ ChIP-seq
☒	☐ Flow cytometry
☒	☐ MRI-based neuroimaging
